# Ukrain – a new cancer cure? A systematic review of randomised clinical trials

**DOI:** 10.1186/1471-2407-5-69

**Published:** 2005-07-01

**Authors:** E Ernst, K Schmidt

**Affiliations:** 1 Complementary Medicine, Peninsula Medical School, Universities of Exeter & Plymouth, 25 Victoria Park Road, Exeter EX2 4NT

## Abstract

**Background:**

Ukrain is an anticancer drug based on the extract of the plant *Chelidonium majus *L. Numerous pre-clinical and clinical investigations seem to suggest that Ukrain is pharmacologically active and clinically effective. We wanted therefore to critically evaluate the clinical trial data in the form of a systematic review.

**Methods:**

Seven electronic databases were searched for all relevant randomised clinical trials. Data were extracted and validated by both authors, tabulated and summarised narratively. The methodological quality was assessed with the Jadad score.

**Results:**

Seven trials met our inclusion criteria. Without exception, their findings suggest that Ukrain has curative effects on a range of cancers. However, the methodological quality of most studies was poor. In addition, the interpretation of several trials was impeded by other problems.

**Conclusion:**

The data from randomised clinical trials suggest Ukrain to have potential as an anticancer drug. However, numerous caveats prevent a positive conclusion, and independent rigorous studies are urgently needed.

## Background

Ukrain (NSC-631570) is a semi-synthetic compound derived from the common weed, greater celandine (*Chelidonium majus *L.). This plant contains a range of alkaloids, most notably chelidonine, also known as benzophenanthridine alkaloid. A leaflet distributed to patients at the Bristol Cancer Help Centre, United Kingdom, describes Ukrain as " the only known product, which at present does not also destroy healthy cells, and which reduces tumors and boosts the immune system..." [1]. Ukrain is most commonly administered intravenously and consists of one molecule thiophosphoric acid conjugated to three molecules of chelidonine. It has drug licenses in several states of the former Soviet Union.

Research on Ukrain started about 20 years ago. Meanwhile, numerous in-vitro studies [2-37] animal experiments [38-83], case reports [84-97], and case series [98-108] have emerged. Collectively, these data suggest that Ukrain has anticancer activity in a wide range of cell lines, which could be of clinical value. Whether or not this translates into clinical effectiveness and whether or not Ukrain does indeed cure some type of cancer or improves their prognosis can best be decided on the basis of randomised clinical trials (RCTs). This systematic review is aimed at summarising and critically evaluating all such studies.

## Methods

Electronic literature searches were conducted in the following databases: MEDLINE (1966 to date, via Pubmed), EMBASE (1974 to date), CINAHL (Cumulative Index to Nursing and Allied Health Literature, 1982 to date), AMED (Allied and Complementary Medicine Database, 1985 to date), PsycINFO (1987 to date), DIMDI (Deutsches Institut für Medizinische Dokumentation und Information) and The Cochrane Central Register of Controlled Trials (CENTRAL). The following search terms were used: 'Ukrain', 'chelidonium', 'greater celandine', 'cancer', 'neoplasm' or 'tumour'. Further handsearches were performed in our unit's own files as well as in the reference lists of all located articles. The producer of Ukrain was also contacted. No restrictions regarding the language of publication were imposed.

We included all RCTs of Ukrain as a treatment for any type of human cancer. Ukrain could be used as a sole treatment or as an adjunct to conventional therapy. Any type of intervention was permitted in the control groups. The clinical endpoints had to be survival or parameters indicative of tumour burden. Non-randomised studies or RCTs that did not quantify clinical endpoints were excluded [e.g. [109-117]], as were duplicates [118].

All articles were read in full by both authors and data relating to design, diagnosis, number of subjects, treatments for experimental and control groups, outcome measures and results were extracted independently by both authors. The methodological quality of each trial was assessed using the Jadad score, unless the study was only available in abstract form [119]. It evaluates methodological quality using three items assessing random allocation, double-blinding and the reports of withdrawals and drop-outs and a maximum of 5 points can be given if all criteria are met. The authors agreed to a consensus on the assessed data and cases of discrepancy would be settled by discussion. Because of overt clinical heterogeneity, a meta-analysis was deemed unreasonable. Descriptive summaries of the data are presented in the following text.

## Results

Our search strategy identified 7 RCTs [120-126]. The majority of these studies was published in two different journals between 1995 and 2002 by 4 different groups of authors from the Belarus and Germany. Key data from these studies are summarised in Table 1 and will be discussed below.

Susak et al published an RCT in which 108 colorectal cancer patients received either Ukrain as a monotherapy or 5-fluororacil for an unspecified time duration [126]. The results suggest that this was followed by non-progression of the malignancy in 88.8% of the patients in the experimental group compared to 27.7% in the control group. This study is only reported in abstract form. Numerous methodological details are therefore not accessible and its methodological quality cannot be reliably assessed.

One year later, the same research group published a similar clinical trial, this time including 96 colorectal cancer patients [120]. Forty-eight patients received Ukrain as a monotherapy and 48 patients received 5-fluorouracil and radiation. The survival rate differed substantially between the two groups. Two-year survival was 78.6% in the experimental group compared to 33.3% in the control group. This study was not blinded but applied an appropriate method of randomisation.

Bondar et al treated 48 histologically verified rectal cancer patients either with X-ray radiotherapy, chemotherapy and surgery (control group) or with Ukrain and surgery (experimental group) [121]. Before and after these treatments, the authors measured 19 different laboratory parameters including two tumour markers. In addition, the Karnofsky Index, tumour dimensions, and recurrences were monitored. All of these variables strongly favoured Ukrain therapy over conventional treatment. This study has, however, numerous limitations. For instance, the method of randomisation was not explained; the authors merely stated that "all patients were subdivided into two randomised groups". Moreover, "tumour dimensions" were mentioned as an outcome measure but neither the methodology of measurement nor the results were provided. The recurrence rates are expressed as percentage figures and no test statistics seem to have been applied.

Uglyanitsa et al conducted a study with 28 patients suffering from bladder cancer [116] aiming "to evaluate the efficacy of Ukrain". Patients were allocated to three groups treated with a total dose of 100 (group 1), 200 (group 2), or 300 mg Ukrain (group 3). Two weeks later tumour regression was verified through cytoscopy and ultrasound. Complete and partial regression was noted in 0/4 patients of group 1, 1/4 patients of group 2, and 2/6 patients of group 3. This study lacks many characteristics of a rigorous trial; its stated aims (to evaluate efficacy) cannot be achieved with the study design, which essentially was that of an equivalence or dose-finding study.

Zemskov and colleagues published a "pilot study" with 42 patients suffering from pancreas cancer who had refused chemotherapy [122]. They were randomised to receive either Vitamin C alone or with Ukrain (total dose 100 mg/patient). The primary endpoint (survival) strongly favoured the Ukrain group. The analysis seems to include 4 protocol violations (the description is unclear). Even though the randomisation procedure is mentioned ('closed envelopes') it seems unusual that precisely 21 patients ended up in both groups. The results are surprisingly good – much better than with any other treatment for that condition.

Uglyanitsa et al randomised ("by lottery") 75 breast cancer patients into three groups of 25 patients each [123]. They received either no specific treatment, a total dose of 50 or 100 mg Ukrain 5–7 days before mastectomy. The authors note that Ukrain rendered the primary tumour and the affected regional lymph nodes larger, harder and "more clearly defined". They interpret this as Ukrain-induced tumour sclerosis. According to the investigators' judgement, these changes facilitated surgery and the operative success. In addition, Ukrain was associated with remarkable symptomatic improvements, e.g. better appetite, more sleep, less weakness. The report is unclear in several respects. For instance, no details about statistical analyses are provided, the outcome measures seem subjective, no information regarding investigator blinding is given, and the randomisation procedure seems suspect.

Zemskov and colleagues randomised 42 patients with pancreatic cancer who had refused conventional therapy [124]. They received either Ukrain (total dose 100 mg/patient) with Vitamin C or Vitamin C alone. The results confirmed this group's earlier findings [122]. Survival was remarkable in the Ukrain treated patients and symptoms responded well to this treatment. There are, however, numerous puzzling details. Why do the authors call their second study a "pilot study"? Why did their ethics committee consent to this "placebo"-controlled trial in the knowledge of the surprisingly positive earlier results? How could a proper randomisation again result in two equally sized groups of 21? In the discussion, the authors describe their earlier results as though this trial was conducted against 5-FU which, in fact, is not the case [122].

Gansauge et al reported a study of 90 patients with pancreatic cancer treated either with 1000 mg gemcitabine/m^2 ^or 100 mg Ukrain or the combination of both regimens [125]. Survival rates suggested that Ukrain was superior to gemcitabine alone. A direct comparison of the 12 month survival rates revealed large differences compared to the data from Zemskov et al [124] (29% vs 76% in the Ukrain-treated groups). The randomisation procedure was not explained and, again, the equal group sizes are remarkable.

## Conclusion

Collectively, these RCTs seem to suggest that Ukrain is an effective therapy for a range of cancers. In conjunction with the numerous encouraging case reports [84-97] case series [98-108], and non-randomised clinical trials [109-121] these data look impressive at first glance. Yet several important caveats need to be considered.

None of the RCTs in this systematic review is without serious methodological limitations. The Jadad score [119] of most RCTs was low. Their sample size was usually small, and a sample size calculation to define the number of patients required was lacking in most cases. Even though most RCTs were non-inferiority studies by design and purpose, their statistical approach was that of a superiority trial. The majority of RCTs were conducted in Ukrainian research institutes and published in only two different journals. In several trials, there are clear signs of involvement of the manufacturer of Ukrain. Most RCTs have generally been poorly evaluated and reported, which possibly reflects the poverty of clinical science in Eastern Europe. Independent replications are not available. The only German study [125] has also been heavily criticised: its sample size (30 patients in each group) is minute, the report lacks statistical detail and there is an inequality of treatment cycles between groups [127]. It was also noted that this study (the only RCT not published in the same two journals as all the other RCTs) was published in a journal for which the senior author served as editor [127]. No RCTs were found showing negative or near neutral results; this might suggest the existence of publication bias for which we did, however, find no definite proof.

Greater celandine (*Chelidonium majus *L), which forms the basis of Ukrain, was traditionally used for liver and gallbladder complaints, loss of appetite and gastroenteritis. None of these indications is supported by trial evidence. The main alkaloid from this plant, chelidonine, has antispasmodic, weak central analgesic and papaverine-like effects. In animal experiments, an alcoholic extract of greater celandine increased bile flow, caused non-specific immune stimulation and acted as a hepatoprotectant [128]. The oral administration of greater celandine in humans has been associated with several cases of toxic hepatitis [129].

The mechanism of action of Ukrain as an anticancer drug (if any) remains elusive. Collectively, the preclinical studies are suggestive of antineoplastic and immunomodulatory effects. It has been postulated that the antineoplastic effect is due to the alkaloids interfering with the metabolism of cancer cells, diminished synthesis of DNA, RNA and proteins, the inhibition of cellular oxygen consumption, and the induction of programmed cell death in malignant cells [130].

Several reports of adverse reactions after greater celandine have been published. Most notably, toxic hepatitis has been associated with its oral use [129,131,132]. No case reports of adverse events have emerged of intravenous Ukrain therapy for cancer. The clinical trial data suggest that Ukrain might cause the following adverse effects: an increase in patients' body temperature (n = 26) [120,123,125], general burning sensations (n = 3) [123] and bleeding (n = 4) [125]. Levels between 0–2 according to World Health Organisation toxicity criteria were noted in two trials [122,124] and toxicity criteria between 0–3 were observed in one trial [125]. The costs of Ukrain therapy are high; one course costs € 700 for the medication alone, and the total treatment costs have been estimated at € 3000 per week [133].

In conclusion, Ukrain is a plant-based anticancer drug that is supported by clinical and pre-clinical evidence in a range of malignancies. The data are, however, not free from problems. Before positive recommendations can be issued, independent replications with definite trials and larger sample sizes seem mandatory.

## Competing interests

The author(s) have no competing interests to declare.

## Authors' contributions

EE conceived of the review, participated in its design and coordination, the data extraction and helped to draft the manuscript. KS carried out the data extraction and helped drafting the manuscript. All authors read and approved the final manuscript.

## Pre-publication history

The pre-publication history for this paper can be accessed here:


